# Preventable Infectious Pathology Dominates Neonatal Readmissions: A Retrospective Analysis from a Pediatrics Department in Ploiești, Romania

**DOI:** 10.3390/children13030330

**Published:** 2026-02-26

**Authors:** Daniela-Eugenia Popescu, Ioana Rosca, Alina Turenschi, Anca Miu, Elena Poenaru, Andreea Teodora Constantin, Gabriel-Petre Gorecki, Leonard Nastase

**Affiliations:** 1 Department of Obstetrics-Gynecology and Neonatology, “Victor Babeș” University of Medicine and Pharmacy, 300041 Timisoara, Romania; popescu.daniela@umft.ro; 2 Department of Neonatology and Department of Pediatrics, Faculty of Medicine and Faculty of Midwifery and Nursing, University of Medicine and Pharmacy “Carol Davila”, 050474 Bucharest, Romania; ioana.rosca@umfcd.ro (I.R.); alina.burcuta@drd.umfcd.ro (A.T.); andreea.constatin@drd.umfcd.ro (A.T.C.); leonard.nastase@umfcd.ro (L.N.); 3 Pediatric Hospital Ploiesti, 100326 Ploiesti, Romania; ancamiu@gmail.com; 4 Neonatology Department, National Institute for Mother and Child Health “Alessandrescu-Rusescu”, 011061 Bucharest, Romania; 5 Faculty of Medicine, Titu Maiorescu University, 031593 Bucharest, Romania; gabriel.gorecki@prof.utm.ro; 6 Department of Anesthesia and Intensive Care, CF 2 Clinical Hospital, 011464 Bucharest, Romania

**Keywords:** neonatal readmission, preventable infections, bronchiolitis, vaccination coverage, Romania, post-discharge care, measles, healthcare quality

## Abstract

**Background:** Readmission of newborns within the first 28 days after initial discharge represents a significant healthcare concern, causing distress to families and financial burden on healthcare systems. Understanding readmission patterns and risk factors is essential for implementing preventive strategies. Objective: This retrospective observational study aimed to evaluate the prevalence and characteristics of neonatal readmissions to a pediatric center in Ploiești, Romania, during a 12-month period (1 January 2024–31 December 2024). **Methods:** We reviewed medical records of all newborns aged 0–28 days admitted to the pediatric hospital after initial discharge from maternity wards. Clinical characteristics, diagnoses, paraclinical findings, and demographic data were analyzed. **Results:** A total of 131 newborns were readmitted, representing a 1.9% readmission rate, and only the first readmission for each patient was included in the analysis. The majority (60.9%) presented with preventable infectious pathology, including bronchiolitis (18.3%), rhinoconjunctivitis (16%), pyoderma (11.4%), infectious gastroenteritis (8.4%), and COVID-19 infection (6.8%). Males comprised 61.3% of cases, and 74.8% were born via cesarean section. Exclusive breastfeeding rate was 45.8%. Concerningly, two cases (1.5%) presented with measles, reflecting declining vaccination coverage in Romania (the lowest in the European Union at 62%). **Conclusions:** The predominance of preventable infectious conditions among neonatal readmissions highlights critical gaps in post-discharge care and infection prevention education. The presence of vaccine-preventable diseases underscores the urgent need to address declining immunization rates in Romania. Enhanced parental education on hygiene practices, infection prevention, and improved post-discharge follow-up systems are essential to reduce neonatal morbidity and readmission rates.

## 1. Introduction

Readmission of newborns within the first month after initial discharge represents a significant healthcare challenge that causes distress to patients and their families, as well as financial pressures on healthcare systems [1]. Research on neonatal readmission has focused on maternal and neonatal factors. Identifying newborns who are at risk for readmission can be useful so that additional support and anticipatory care can be provided to at-risk families [1,2]. Approximately 3 to 7 percent of newborns are readmitted to the hospital within 30 days after discharge from maternity. Common reasons for readmission include feeding-related difficulties and hyperbilirubinemia, while other risk factors for newborn readmission are lower gestational age, premature discharge, young and/or inexperienced parents, difficulties in establishing infant feeding during hospitalization at birth, and presence of one or more risk factors for severe or progressive hyperbilirubinemia [2,3].

Newborns may acquire an infection through vertical transmission from the mother during delivery or postnatally from sources such as hospital personnel, family members, and daycare staff or attendees [4,5,6]. Neonates are more likely than older infants to experience morbidity from a viral infection, in part because of a decreased responsiveness of T-cell-mediated immunity [7,8,9]. Thus, viral infections in newborns should be prevented, as morbidity and mortality in this age group is increased [10]. Standard strategies to reduce the risk of infections include appropriate hand hygiene, such as washing with soap or using alcohol-based hand rubs, to limit the transmission of infectious agents. Additional preventive measures include minimizing passive exposure to cigarette smoke and avoiding contact with individuals with respiratory tract infections [11,12]. Also, specific strategies to prevent severe RSV infections in infants include vaccination of the mother who gave birth to the child during pregnancy and immunoprophylaxis with monoclonal antibodies [13]. Humanity has gone through a COVID-19 pandemic, and for newborns, this may be another viral cause manifested both in the perinatal period and after discharge from the maternity hospital [14,15]. Current evidence suggests that breast milk is not a source of transmission of SARS-CoV-2, and when possible, breastfeeding mothers with suspected or confirmed COVID-19 should continue to breastfeed while taking hygiene precautions (consider wearing a mask during any close contact with the child, frequent handwashing, improving ventilation indoors by opening windows, using air filters, and turning on fans). This advice is regardless of COVID-19 vaccination status [16].

The neonatal period is important for establishing a strong healthy foundation and is also associated with high mortality and morbidity rates. As neonatal readmission rates can reach 10.1% outside the US, this is a global concern. Neonatal readmissions impose significant costs on patients, their families, and the healthcare system as a whole. A number of tests and examinations are performed to determine whether a newborn is ready for discharge from maternity units. However, some newborns may experience readmission at any age. For this reason, determining associated maternal and neonatal risk factors is crucial when considering how age affects neonatal outcomes, caregivers, and financial obligations [17,18,19].

In general, in Romanian maternity hospitals, newborns are discharged at 72 h of life if born by cesarean section or at 48 h of life if born vaginally. If the mother–newborn dyad meets the criteria for discharge, discharge can be earlier than 72 h (i.e., the newborn is breastfeeding, blood tests and clinical status are normal, and the mother has been informed and trained on care and resolution of any problems that may arise at home) [20,21]. There is also a constant concern of neonatologists to provide support to parents through follow-up in maternity hospitals 7–10 days after discharge or whenever they need it during the period of 3–28 days of life to decrease the rate of readmission to pediatrics [22].

## 2. Materials and Methods

We conducted a retrospective observational cohort study that aimed to evaluate the prevalence of newborns in a pediatric center in Ploiesti, Romania, investigating discharged newborns (0–28 days) born from maternity and admitted to Ploiesti Pediatric Hospital over a period of twelve months (1 January 2024–31 December 2024). In this study, “readmission” was defined as any hospitalization in the pediatric department within the first 28 days of life occurring after discharge from a maternity unit, regardless of the birth hospital. The data was collected by the authors in Microsoft Office Excel 2013 from the electronic medical records of the hospital and double-checked. The data collected included pregnancy information (mode of conception, gestational age at birth, APGAR score and type of delivery), patient demographics (age, sex, method of feeding), clinical data (length of stay in the pediatric unit and diagnosis at admission), and laboratory tests (cultures, CRP, leukocyte count, PCR, serology). Diagnoses were established based on clinical presentation, laboratory findings, and microbiological results, in accordance with routine neonatal clinical practice. Late neonatal sepsis was defined by the presence of clinical signs suggestive of systemic infection (such as fever/hypothermia, apnea, feeding intolerance, lethargy, or respiratory distress), associated with abnormal inflammatory markers (elevated C-reactive protein, abnormal leukocyte count, procalcitonin and presepsin), and/or positive microbiological cultures when available. In cases with negative cultures, the diagnosis was based on the combination of clinical deterioration and laboratory evidence of infection.

**Figure 1:**
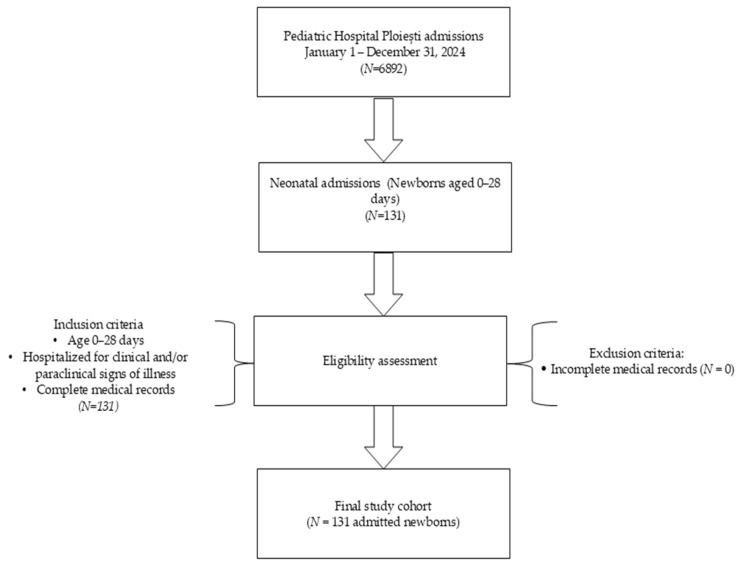
Flowchart of study design and patient selection.

The inclusion criteria were all newborns with signs of illness hospitalized in pediatrics aged up to 28 days, based on clinical or paraclinical data. Exclusion criteria were incomplete data (no patient met the exclusion criteria (Figure 1)). Only the first readmission episode per newborn within the first 28 days of life was included in the analysis. Repeated readmissions of the same patient were not analyzed separately.

## 3. Results

Our study included 131 newborns admitted to the pediatric hospital in Ploiesti who were discharged from the maternity ward in good general condition but in the immediate period following, up to 28 days of life, they presented to the pediatric emergency room with varied symptoms, specific to the newborn but also to the young child.

### 3.1. Patient Characteristics

Of the 131 readmitted neonates, 79 (61.3%) were male and 52 (39.7%) were female. The majority, 123 (93.9%), were conceived naturally, while 8 (6.1%) resulted from IVF. Regarding gestational age and birthweight, 106 (80.9%) were term newborns with normal birthweight, 19 (14.5%) were late preterm infants (35–36 weeks), and 6 (4.5%) were term with intrauterine growth restriction (IUGR).

APGAR scores were favorable in most cases: 118 (90.1%) had scores of 8–10, while 13 (9.9%) had scores of 6–7; no newborn had a score below 5. Cesarean section was the predominant mode of delivery, accounting for 98 (74.8%) of cases, while only 33 (25.1%) were born vaginally.

Regarding hospital length of stay, 74 (56.4%) were hospitalized for 5 days or less, while 57 (43.5%) required more than 5 days. For feeding method, 60 (45.8%) were exclusively breastfed, 50 (38.1%) received mixed feeding (breast milk and formula), and 21 (16%) were formula-fed only.

The age at admission was distributed as follows: 9 (6.8%) between 2–7 days, 47 (35.8%) between 8–14 days, 38 (29%) between 15–21 days, and 37 (28.2%) between 22–28 days of life. A total of 19 (14.5%) were late preterms (35–36 weeks), and the other 112 (84.8%) were at-term newborns (Table 1).

### 3.2. Diagnoses at Admission

The most common diagnosis was bronchiolitis, affecting 24 (18.3%) patients, followed by rhinoconjunctivitis in 21 (16%), pyoderma in 15 (11.4%), diarrhea and infectious gastroenteritis in 11 (8.4%), and COVID-19/SARS-CoV-2 infection in 9 (6.8%). Apnea was observed in nine (6.8%) cases, gastroesophageal reflux in seven (5.3%), late neonatal sepsis in six (4.5%), and urinary tract infection in six (4.5%). Less common diagnoses included febrile syndrome in five (3.8%), vomiting in five (3.8%), jaundice in three (2.2%), protein–energy malnutrition in three (2.2%), and measles in two (1.5%) cases. Single cases (0.76% each) were recorded for hypertrophic pyloric stenosis, neonatal seizures, whooping cough (pertussis), hypothyroidism, and omphalitis (Table 2).

### 3.3. Paraclinical Evaluation

Umbilical swab cultures revealed methicillin-resistant Staphylococcus aureus (MRSA) in eight cases, Staphylococcus aureus in two, and Klebsiella in three. Nasal swab cultures identified Staphylococcus aureus in 23 cases and Proteus in 2. One ophthalmic swab was positive for Staphylococcus aureus. Urine cultures showed Klebsiella pneumoniae in two cases and Escherichia coli in four cases. Viral testing revealed 9 positive COVID-19 antigen/PCR results, 1 positive Type A Influenza antigen, 12 positive RSV antigen results, and 7 positive Rotavirus antigen results. A total of 9 patients had elevated CRP (>5 mg/dL), 19 had anemia (Hb < 12 g/dL, Ht < 35%), 3 had hyperbilirubinemia (total serum bilirubin > 10 mg/dL), 2 had hypocalcemia, and 1 had LDL cholesterol > 130 mg/mL. Serology confirmed two measles cases, one pertussis case, and one hypothyroidism case (Table 3).

## 4. Discussion

Our study examined neonatal readmission rates in the first 3 and 28 days of life between January and December 2024. We report a newborn admission rate of 1.9% in the first 28 days of life over the one-year study period. This lower readmission rate coincided with an increase in total admissions, from 5226 (2022–2023) to 6892 (2024) [22]. This indicates an improvement in the care and management of newborns. Indeed, this is lower than the readmission rate in a Canadian study that reported a readmission rate of 4% [23]. Although preterm infants theoretically have higher readmission rates], the low proportion of preterm in our cohort (14.5%) and their late preterm gestational age (35–36 weeks) can be explained by two considerations. First, very preterm infants are more frequently followed-up in specialized maternity units in the capital city of Bucharest, located approximately 80 km away. Second, this study included only patients up to 28 days of life, and very preterm infants typically spend these days hospitalized in the maternity unit, being discharged later [24,25,26].

More than half of the readmitted patients (60.9%) presented with preventable infectious pathology (bronchiolitis, acute rhinoconjunctivitis, pyoderma, diarrhea and infectious gastroenteritis, COVID-19/SARS-CoV-2 infection), representing the main reasons for readmission. Bronchiolitis, the leading cause of readmission in our cohort (18.3%), is an acute inflammatory injury of the bronchioles that primarily affects infants and young children under 2 years of age, with peak incidence occurring between 2 and 6 months of age, RSV being the most common cause [27,28]. The neonatal period represents a particularly vulnerable time, as young infants are at increased risk for severe disease due to their immature immune systems and smaller airways [29]. COVID-19 infection affected nine neonates (6.8%) in our study. SARS-CoV-2 infection in neonates can manifest with varied clinical presentations, from mild respiratory symptoms to more severe systemic involvement [30,31,32]. Infectious gastroenteritis (8.4%) and rotavirus infection (seven positive antigen results) represent significant causes of morbidity in our cohort. Rotavirus is the leading cause of severe gastroenteritis in children under 5 years of age, continuing to result in more than 200,000 deaths worldwide each year [33]. The burden of gastrointestinal infections in newborns is generally reduced due to protective factors such as exclusive breastfeeding; however, rotavirus and pathogenic strains of E. coli remain the main etiological agents [34,35].

Two neonates (1.5%) presented with measles and one with pertussis, highlighting the alarming consequences of declining vaccination coverage in Romania [36,37,38]. These findings are deeply concerning given the severe complications associated with these infections in the neonatal period. Romania has the lowest measles vaccination coverage in the European Union (EU), with only 62% of people fully immunized as of 2023, far below the 95% threshold needed for herd immunity [39,40]. In 2024, Romania reported around 30,000 measles cases, being the highest notification rate in the EU at 1610.7 cases per million population (87% of all EU cases) [40]. Moreover, 18 of the 19 measles-related deaths in the EU occurred in Romania [40]. The presence of measles in neonates is alarming due to the risk of subacute sclerosing panencephalitis (SSPE), a rare fatal neurological complication of measles infection that typically manifests 7–10 years after the initial infection, with shorter latency periods observed in children infected before age 2 [41,42]. The risk of developing SSPE is highest among infants: while the general risk is 4–11 per 100,000 measles cases, it is estimated that for unvaccinated infants under 15 months, the risk was as high as 1 in 600 [42,43,44]. SSPE is an incurable disease with up to 95% mortality rate, vaccination being the only efficient method to combat it [45,46]. The last epidemiological data on SSPE in Romania dates back to 1976–1982, but given the current massive measles outbreak and low vaccination rates, particularly affecting infants who cannot yet be vaccinated, there is a substantial risk of SSPE re-emergence in Romania in the coming years. The two neonates with measles in our study represent not only an immediate health crisis but also a potential long-term tragedy, as these children may develop SSPE years later [47,48].

Pertussis represents another vaccine-preventable disease in our cohort, with one affected neonate. Europe has experienced a surge of pertussis cases from 2022–2024, with nearly 60,000 cases reported by April 2024 [49,50]. Infants under six months, unimmunized or partially immunized, face the highest risk of severe outcomes, with the majority of pertussis-related hospitalizations and deaths occurring in this vulnerable age group [51]. Romania has contributed to these alarming European trends, with recent studies documenting the re-emergence of pediatric pertussis as a significant public health concern [51,52,53]. Numerous factors have been identified as contributing to Romania’s declining vaccination coverage, including vaccine hesitancy, nonattendance at follow-up appointments, especially in rural communities, missed doses due to temporary medical contraindications, population shifts that result in children missing follow-up appointments, a decline in the number of general practitioners, discontinuities in vaccine supply, and widespread anti-vaccine misinformation that has been exacerbated by the COVID-19 pandemic [54,55]. Additionally, hepatitis B vaccination coverage, while historically successful in reducing incidence from 43 per 100,000 in 1989 to 8.5 per 100,000 in 2004, has experienced concerning declines in recent years, with Romania reporting one of the largest decreases (3%) in the EU [56,57].

Pyoderma caused by MRSA and Staphylococcus aureus accounted for a notable proportion of cases (11.4%). This finding may be associated with gaps in infection prevention practices during hospitalization and/or after discharge. Children of all ages, including neonates, can become infected with MRSA. Epidemiological risk factors associated with the spread of MRSA in the child or family include close skin-to-skin contact, openings in the skin such as cuts or abrasions, contaminated items and surfaces, crowded living conditions and poor hygiene [58,59,60].

A low percentage of patients (2.2%) presented with jaundice and protein–energy malnutrition, which indicates both prompt and correct management in the maternity ward and correct feeding at home. A study from Italy for term infants with early discharge and a follow-up program after discharge found no readmissions for jaundice or dehydration in the first 28 days of life [61]. One of these newborns presented multiple pathologies within Down’s syndrome: jaundice, hypothyroidism, and cardiac malformation. Specialists in neonatology and pediatrics anticipated this multiple pathology and called on the multidisciplinary team [62,63,64]. One of the patients with jaundice and protein–energy malnutrition presented with laboratory tests showing LDL cholesterol > 130 mg/mL, which required interdisciplinary consultation and genetic testing; clinical signs are rare in pediatric patients, and even though these disorders are rare, there are new studies that encourage screening of lipid disorders in newborns [65,66,67].

Cesarean section rates were much higher (74.8%) than vaginal birth rates (25.1%) among infants readmitted in our study. Infants born by cesarean section are more likely to be discharged at a postnatal age of 3–4 days, which explains the lower rate of protein–energy malnutrition, better-educated mothers, and the use of formula milk in this category of newborns. Our study results are similar to those of a population-based cohort study in Canada, which observed higher rates of cesarean delivery among readmitted infants, although our country has a higher rate of cesarean versus vaginal births [17]. Romania has one of the highest C-section rates in the world, being reported as the third highest rate country in Europe in 2021 (44.3%) [68,69].

We observed low rates of exclusive breastfeeding (45.8%) in infants with readmissions. This finding is consistent with the international literature demonstrating that exclusive breastfeeding rates are often suboptimal among readmitted infants. Studies have shown that the readmission rate for breastfeeding problems ranges from approximately 0.3% to 2% of term or near-term infants [70]. Breastfeeding problems should be anticipated and assessed before discharge from the maternity ward, with appropriate lactation support provided to at-risk mother–infant dyads.

The predominance of preventable infectious conditions (60.9%) in our cohort necessitates a structured prevention structure. We propose two main prevention categories based on our findings.

### 4.1. Vaccine-Preventable Diseases

The two measles cases (1.5%) and one pertussis case (0.76%) in our study reflect the urgent need for vaccination interventions. Maternal immunization during pregnancy, particularly the Tetanus, Diphtheria, and Pertussis (Tdap) vaccine at 27–36 weeks of gestation and the RSV vaccine at 32–36 weeks of gestation, provides passive immunity to newborns during their most vulnerable period [13,49]. For RSV prevention, which contributed to 18.3% of readmissions (through bronchiolitis), both maternal vaccination and nirsevimab (monoclonal antibody prophylaxis) have shown efficacy [14]. Newborn vaccination schedule adherence requires systematic follow-up, including reminder systems via SMS or phone calls by the general practitioner (GP) to ensure completion of primary immunizations [55,56]. Addressing vaccine hesitancy through evidence-based parental counseling before discharge is essential, particularly given Romania’s low vaccination coverage [39,40].

### 4.2. Basic Medical and Hygiene Education

Pyoderma (11.4%), rhinoconjunctivitis (16%), and MRSA colonization (eight cases) may indicate gaps in infection prevention practices. Appropriate parental education regarding basic hygiene practices is essential not only for infection prevention but also for recognizing signs of potential child abuse or neglect, which represents a broader concern in pediatric care [71]. Hand hygiene education is fundamental, with demonstrated efficacy in reducing pathogen transmission when performed correctly using soap and water or alcohol-based hand rubs [11,12]. Pre-discharge structured training should include the WHO six-step handwashing method, with emphasis on timing (before feeding, after diaper changes). Environmental hygiene counseling should address proper umbilical cord care to prevent omphalitis, skin care to reduce pyoderma risk, and limiting exposure to individuals with infections [4,6]. Hospital infection control protocols must be strengthened to reduce MRSA transmission, particularly in maternity wards where colonization may occur [59,60,61].

Effective prevention requires both offline education (pre-discharge structured sessions covering infection signs recognition, feeding support, and hands-on demonstrations of proper hygiene practices) and online platforms (mobile applications with vaccination reminders, telemedicine support hotlines for parental concerns, and social media campaigns to combat misinformation) [23,62]. Studies demonstrate that early post-discharge follow-up (3–7 days) combined with accessible healthcare support reduces readmission rates [2,62]. Community-level interventions, including home visits for high-risk families and peer support programs, further strengthen prevention efforts. This integrated approach addresses the gaps identified in our cohort, where exclusive breastfeeding was only 45.8% and preventable infections dominated readmissions. Another important form of education should be the implementation of educational and supports programs for the parents of children with chronic disease with early onset, such as cystic fibrosis, chronic liver and biliary diseases, HIV infection or cerebral palsy [72,73,74,75,76].

The implementation of vaccination and medical education could significantly reduce readmissions in the neonatal period and even in further periods. Success requires coordinated efforts across prenatal care, maternity wards, primary care, and public health programs, with particular emphasis on addressing Romania’s vaccination coverage crisis.

## 5. Conclusions

This study demonstrates that preventable infectious pathology dominates neonatal readmissions at our center, accounting for over 60% of cases. The findings highlight several critical areas requiring intervention. Infection prevention education should be strengthened before discharge and during post-discharge follow-up, emphasizing hand hygiene, avoiding contact with sick individuals, and recognizing early warning signs of infection. Vaccination programs require urgent reinforcement. The presence of measles and pertussis in our neonatal cohort reflects Romania’s critically low vaccination coverage—the lowest in the EU. Breastfeeding support systems need enhancement to improve exclusive breastfeeding rates and reduce feeding-related complications. Hospital infection control measures require ongoing vigilance, particularly regarding MRSA and other healthcare-associated pathogens.

## Figures and Tables

**Table 1:** Patients characteristics.

Characteristics	Number of Patients (*n* = 131)
Gender	
• Male	79 (60.3%)
• Female	52 (39.7%)
Mode of conception	
• Natural	123 (93.9%)
• IVF	8 (6.1%)
Gestational age and birthweight	
• At term with normal birthweight	106 (80.9%)
• Preterm	19 (14.5%)
• At term with IUGR	6 (4.6%)
APGAR score	9 (9–10)
• 8–10	118 (90.1%)
• 6–7	13 (9.9%)
• <5	0
Type of delivery	
• Vaginal	33 (25.2%)
• Caesarian	98 (74.8%)
Length of stay in the hospital (pediatric unit)	
• ≤5 days	74 (56.5%)
• >5 days	57 (43.5%)
Method of feeding	
• Breastfed	60 (45.8%)
• Mixed feeding (breastfed and formula)	50 (38.2%)
• Only formula	21 (16%)
Newborn age at admission	
• 2–7 days	9 (6.9%)
• 8–14 days	47 (35.9%)
• 15–21 days	38 (29%)
• 22–28 days	37 (28.2%)

IUGR (intrauterine growth restriction). IVF (in vitro fertilization).

**Table 2:** Diagnoses of patients included in the study (131).

Diagnosis at Admission	Number of Patients Diagnosed (*n* = 131)
Bronchiolitis	24 (18.3%)
Rhinoconjunctivitis	21 (16%)
Pyoderma	15 (11.4%)
Diarrhea and infectious gastroenteritis	11 (8.4%)
COVID-19/SARS-CoV-2 infection	9 (6.8%)
Apnea	9 (6.8%)
Gastroesophageal reflux	7 (5.3%)
Late neonatal sepsis	6 (4.5%)
Urinary tract infection	6 (4.5%)
Febrile syndrome	5 (3.8%)
Vomiting	5 (3.8%)
Jaundice	3 (2.2%)
Protein–energy malnutrition	3 (2.2%)
Measles	2 (1.5%)
Hypertrophic pyloric stenosis	1 (0.76%)
Neonatal seizures	1 (0.76%)
Whooping cough (Pertussis)	1 (0.76%)
Hypothyroidism	1 (0.76%)
Omphalitis	1 (0.76%)

**Table 3:** Paraclinical evaluation results in admitted patients.

Investigation and Result	Number of Patients
Umbilical swab	
• MRSA	8
• Methicillin-sensible Staphylococcus aureus (MSSA)	2
• Klebsiella	3
Nasal swab	
• Staphylococcus aureus	23
• Proteus	2
Ophthalmic swab–Staphylococcus aureus	1
Urine culture	
Klebsiella pneumoniae	2
Escherichia coli	4
Positive COVID-19 antigen/PCR	9
Positive Type A Influenzae antigen	1
Positive RSV antigen	12
Positive Rotavirus antigen	7
High CRP (C reactive protein) (>5 mg/dL)	9
Complete blood count revealing anemia (Hb < 12 g/dL, Ht < 35%)	19
Hyperbilirubinemia (total serum bilirubin > 10 mg/dL)	3
Hypocalcemia	2
LDL cholesterol > 130 mg/mL	1
Measles serology	2
Pertussis serology	1
Hypothyroidism	1

RSV (respiratory syncytial virus). MRSA (methicillin-resistant Staphylococcus aureus). MSSA (methicillin-sensible Staphylococcus aureus).

## Data Availability

Data are contained within the article.

## Abbreviations

The following abbreviations are used in this manuscript:

IUGR	Intrauterine growth restriction
IVF	In vitro fertilization
RSV	Respiratory syncytial virus
MRSA	Methicillin-resistant Staphylococcus aureus
